# Spatio-Temporal Variation in Water Uptake in Seminal and Nodal Root Systems of Barley Plants Grown in Soil

**DOI:** 10.3389/fpls.2020.01247

**Published:** 2020-08-13

**Authors:** Hannah M. Schneider, Johannes A. Postma, Johannes Kochs, Daniel Pflugfelder, Jonathan P. Lynch, Dagmar van Dusschoten

**Affiliations:** ^1^Forschungszentrum Jülich, IBG-2, Jülich, Germany; ^2^Department of Plant Science, The Pennsylvania State University, University Park, PA, United States

**Keywords:** barley, MRI, nodal, seminal, root, water uptake

## Abstract

The spatial and temporal dynamics of root water uptake in nodal and seminal roots are poorly understood, especially in relation to root system development and aging. Here we non-destructively quantify 1) root water uptake and 2) root length of nodal and seminal roots of barley in three dimensions during 43 days of growth. We developed a concentric split root system to hydraulically and physically isolate the seminal and nodal root systems. Using magnetic resonance imaging (MRI), roots were visualized, root length was determined, and soil water depletion in both compartments was measured. From 19 days after germination and onwards, the nodal root system had greater water uptake compared to the seminal root system due to both greater root length and greater root conductivity. At 29 days after germination onwards, the average age of the seminal and nodal root systems was similar and no differences were observed in water uptake per root length between seminal and nodal root systems, indicating the importance of embryonic root systems for seedling establishment and nodal root systems in more mature plants. Since nodal roots perform the majority of water uptake at 29 days after germination and onwards, nodal root phenes merit consideration as a selection target to improve water capture in barley and possibly other crops.

## Introduction

Suboptimal water availability is a primary limitation to crop productivity worldwide (Forster et al., 2004). Plant roots play a key role in soil water acquisition and drought tolerance and influence plant adaptation to a variety of environmental conditions (McCully, 1999; Javaux et al., 2008; Chloupek et al., 2010; Lynch, 2013). Root water uptake controls the dynamics of water flow, soil moisture, and solute transport in the root zone and rhizosphere; however, little is known about the effects of root class and anatomy on water uptake.

Root water uptake patterns are determined by a variety of factors including the interaction between the spatial and temporal placement of roots in the soil, the distribution of water in the soil, and the hydraulic conductance of both the seminal and nodal root systems. Root classes differ in size, anatomy, and architecture and therefore may have large effects on water uptake. The barley root system consists of two major root classes: seminal axial (or seed-borne) and nodal axial (shoot-borne) roots which all produce lateral (root-borne) roots of the first and second order. The seminal root system consists of several seed-borne roots that emerge nearly simultaneously at the beginning of plant growth. Later in development, the post-embryonic nodal roots form and continue to develop throughout plant vegetative growth and tillering stages (Hecht et al., 2019). Root class, architecture, and anatomy determine the spatial and temporal distribution of roots in specific soil domains and their ability to obtain mobile and immobile resources (Lynch, 1995; Hirel et al., 2007; Lynch and Brown, 2012; Lynch, 2013; Lynch, 2019). Root traits that enable the exploration of deep soil domains influence the capture of mobile soil resources, like water, as mobile soil resources are generally more available in deeper soil domains over time due to crop uptake, evaporation, and leaching throughout the growth season (Lynch and Wojciechowski, 2015).

Root class is thought to be an important factor in root water uptake, but to date few studies have provided quantitative data. Studies of 16 day old maize plants showed that water uptake of lateral roots was substantially greater than water uptake of segments of seminal roots due to a greater uptake per root length (Ahmed et al., 2016) which may be important as the metabolic costs of construction and maintenance of lateral roots differ from axial roots (Zhan et al., 2015). Similarly, in roots of lupine plants, water uptake was not uniform along the roots, and water uptake was greater in the shallow soil layers compared to deeper soil domains. The majority of water uptake in lupine occurred in lateral roots, and radial flux was greater in proximal (located adjacent to the base of the stem) compared to distal segments (near the root tip) (Zarebanadkouki et al., 2013). In citrus, axial roots had a significantly greater water uptake rate compared to first and second order lateral roots (Rewald et al., 2011). In young barley plants (14–17 DAG), seminal roots contributed more root water uptake compared to nodal roots due to both a greater root length and root hydraulic conductance (Knipfer and Fricke, 2011). However, water uptake by lateral roots may be more important in maintaining plant water status compared to root water uptake by the main axis roots as barley lateral roots contribute between 25 and 60% of total root water uptake (Graham et al., 1974; Sanderson, 1983). These studies highlight the importance of evaluating root water uptake in different root classes and to what extent differences in root size and anatomy play a role in order to develop improved ideotypes for water uptake.

The location of water transport into the root depends on a variety of factors including the hydraulic conductivities of the root–soil interface, the radial path from the root epidermis to the xylem, and the axial path through the xylem (Steudle and Peterson, 1998). Hydraulic conductivity varies during root maturation and in response to environmental conditions, primarily due to varying aquaporin-mediated water transport and developmental changes in root anatomy (Adiredjo et al., 2014; Hu et al., 2014; Grondin et al., 2015; Schneider et al., 2017b). Aquaporins can contribute up to 90% of root hydraulic conductivity in barley (Knipfer et al., 2011). In rice, aquaporins are differently regulated in edaphic stress conditions (Grondin et al., 2015). Anatomical modifications of the root tissue due to root maturation and/or environmental conditions may decrease root hydraulic conductivity (Steudle and Peterson, 1998; Enstone et al., 2003; Bramley et al., 2009). Differentiation of metaxylem vessels along a single root axis affects axial conductivity (Varney and Canny, 1993; McCully, 1995; Bramley et al., 2009), and the development of suberized cell layers in the exodermis or endodermis may decrease radial hydraulic conductivity (Ranathunge et al., 2017). One of the factors that may greatly affect radial hydraulic conductance is change to the root cortex. The development of root cortical senescence in barley and root cortical aerenchyma in maize, which both involve programmed cell death of cortical cells, has been demonstrated to reduce radial conductivity and transport of water and nutrients through the cortex (Fan et al., 2007; Hu et al., 2014; Schneider et al., 2017b). This reduction in radial conductivity due to root cortical senescence occurs despite the loss of the epidermal and cortical cells, which are normally considered a barrier. The root cross-sectional area declined by approximately 56% after 30 days of growth (Schneider et al., 2017b).

Studies on the spatial distribution of water uptake show that roots grown in solution or aeroponic cultures have distinctly different properties than roots grown in soil, and in contrast, field grown plants and measurements do not allow for detailed, non-destructive physiological measurements on water dynamics. In addition, in drier soils, soil water conductivity may be the main determinant of root water uptake (Hayat et al., 2019). The majority of experimental data on the spatial distribution of water uptake is limited to roots grown in solution and aeroponic cultures (Frensch and Steudle, 1989; Varney and Canny, 1993; Zwieniecki et al., 2002) which have distinctly different properties than roots grown in soil (Varney and Canny, 1993). The quantification of water uptake *in situ* from roots grown in soil is challenging; however, it addresses important factors including the root–soil contact which is an important driver of hydraulic conductivity at the root–soil interface (Carminati et al., 2012; Zarebanadkouki et al., 2014). The development of non-invasive two- and three- dimensional methods has enabled the quantification of root water uptake with high spatial and temporal resolution. MRI technology enables non-destructive three-dimensional imaging of structures and transport processes in porous media (Van As and van Dusschoten, 1997; Gregory and Hinsinger, 1999). MRI functions by manipulating the magnetic moment of atomic nuclei (like ^1^H protons), that are abundant in living tissues, through strong magnetic and radio frequency fields. An application of magnetic resonance, diffusion tensor imaging, enabled the creation of a spatial map of water mobility in a pot with roots and soil (Gruwel, 2014). These technological advancements can be used to correlate root development and architecture to spatial and temporal dynamics of root water uptake.

MRI technology enables quantification of soil water and root length and diameter non-destructively. MRI is a nuclear specific volumetric imaging modality that can visualize structures within opaque media with adjustable contrast capabilities (Callaghan, 1993). Here, we use this capability to visualize water in roots to measure root length and diameter (van Dusschoten et al., 2016) and, using different contrast settings, soil water content (*e.g.* Pohlmeier et al., 2008). A simple calibration value can be used to quantify the amount of water per volumetric unit such that root diameter can be determined within the soil (van Dusschoten et al., 2016). Similarly, soil water content can be quantified using calibration curves (Haber-Pohlmeier et al., 2010).

We ask if the seminal and nodal root systems differ in root length development and water capture throughout plant growth using barley (*Hordeum vulgare*) as a model system. First, we characterized and quantified root signal intensity changes to enable the accurate estimation of root length, as the root signal intensity can vary throughout plant growth. With MRI, we quantified the degree of root cortical senescence formation as it is an important factor in radial hydraulic conductivity. In addition, we used microscopy at harvest to phenotype other anatomical phenes to estimate axial hydraulic conductivity. Second, we developed a concentric split root system that physically and hydraulically separates the nodal and seminal root system. A concentric split root system enables the measurement of the seminal and nodal root systems separately. This split root system was used to perform specific water uptake studies on the seminal root system (seminal root axes including their laterals) and nodal root system (nodal root axes including their laterals) using three-dimensional MRI sequences optimized to either detect soil water or roots. An 80 cm pot (80 cm in height, 8 cm radius) enabled roots to grow relatively unimpeded by pot size or depth for 43 days. Given both compartments were maintained at the same high moisture content, water depletion was used to study the relative water uptake and relative conductivity of the seminal and nodal root systems. This approach facilitates the quantification of water uptake and root length of nodal and seminal root systems separately in barley spatially and temporally during 43 days of growth. Understanding spatial and temporal root and water dynamics and how root class influence root water uptake is important to gain insights on plant function and adaptation. Insights into plant water uptake will be an important consideration as a selection target to improve water capture in barley.

## Materials and Methods

### Plant Material and Growth Conditions

#### Root Water Uptake Experiments in a Cylindrical Split-Root System

Two barley genotypes (Tkn24b and Arena, seeds obtained from IPK Gatersleben, Germany) that vary in root anatomical traits were grown in two replications (*i.e.* four plants totally grown in four individual split root system pots). Seeds were surface sterilized in 1.5% NaOCl in water and rolled into tubes of germination paper (76 lb, Anchor Paper, St Paul, MN, USA). Rolls were placed vertically in covered beakers containing 0.5 mM CaSO_4_ in a dark climate chamber at 28°C for 4 days. Afterwards, the beakers containing germinated seedlings in rolls were placed under a constant fluorescent light (350 µE m^−2^s^−1^) at 28°C for one day in a climate chamber. Seedlings were transplanted into the MRI concentric split root system (**Supplemental Figure 1**). In short, the inner ring (height 72 cm, diameter 5.4 cm) was fitted with a plastic funnel (length 6 cm, start width 6.2 cm, exit width 1.2 cm) on top and placed inside the outer ring (height 80 cm, diameter 8 cm). A fitted base kept the tubes in place. Results from pilot experiments on barley plants grown in relatively shallow mesocosms (30 cm) demonstrated that the use of deeper mesocosms is necessary as the seminal root system reached the bottom of the mesocosm within one week of growth. Once plant roots reach the bottom of the mesocosm, their anatomy and growth are affected, so we chose a mesocosm size (80 cm) that would allow us to grow the plants for 43 days with minimal mesocosm interference. With this setup, seminal and nodal roots did not reach the bottom of the pot until 43 DAG. The inner and outer compartments were filled with MRI compatible loamy sand soil (van Dusschoten et al., 2016) to a dry bulk density of 1650 kg m^−3^ (g of soil per volume of pot compartment) until the top of the funnel. In brief, sandy loam soil from an agricultural field was sieved to 2 mm and mixed with coarse sand (1:2, v/v). Further soil preparation and property details are described in van Dusschoten et al., 2016. The germinated seedling (with all of the seminal roots emerged, no nodal roots emerged) was placed 1 cm above the top of the funnel such that the seminal roots were inside the inner tube/funnel, but the seed remained 1 cm above the funnel top. This enabled the seminal roots to grow in the inner compartment and the nodal roots to develop in the outer compartment (**Supplemental Figure 1**). Nodal roots began to emerge at 14 DAG, and all grew in the outer compartment. The rest of the mesocosm was filled with soil to the same bulk density. The inner and outer compartments were watered to 12% soil water (by volume, outer compartment: 2,664 cm^3^, inner compartment: 1,357 cm^3^). Mesocosms were transferred to a climate chamber (22/16°C, 14 h daylight, 50% RH). Water content in the inner and outer compartments was measured individually every other day for the first week of plant growth and then daily until the end of the experiment. Water content was measured in the inner tube using a home-built, detachable capacitive sensor inserted at the bottom of the mesocosm with an accuracy of +/−2% soil water content. Water content in the outer compartment was calculated *via* the weight of the mesocosm after correction of the amount of water in the inner compartment. Soil water content was maintained at 12% or lower. It is important to keep the water concentration the same and preferably high in the inner and outer compartments so that the seminal and nodal roots develop at similar soil water potentials. Also, it minimizes preferential water uptake from the wetter compartment. Keeping the water content below 12% prevents overwatering (*i.e.* above field capacity), which may cause water to drain out of the bottom of the mesocosm, a factor that cannot be distinguished from root water uptake in the MRI. The inner and outer compartments were maintained at ~12% soil water content and were irrigated from the top of the pot. Fertilizer was applied 1 day after transplant (0.5% Hakaphos blue stock solution prepared according to manufacturer instructions, Compo, Münster, Germany, 25 ml per plant). Root systems and soil water content were measured in the MRI at 19, 29, 34, and 43 days after germination (DAG). Plants were measured weekly for plant height and tiller number.

#### Experiments to Relate MRI Signal Intensity to Root Age

In a supplementary experiment, barley seeds (genotypes Arena and Tkn24b) were directly sown in eight replications (16 pots total) into mesocosms (40 cm × 8 cm) filled to a bulk density of 1,650 kg m^−3^ with MRI compatible soil (details above). The purpose of this experiment was to quantify and characterize MRI signal intensity loss in relation to root age to enable the accurate estimation of root length. Pilot experiments demonstrated that the root signal intensity can vary throughout plant growth and we wanted to accurately estimate root length in the MRI. Mesocosms were placed in a climate chamber (details above). Soil water content was maintained at 12% and measured daily using an MRI compatible capacitive sensor (details above). At 12, 19, 27, and 35 DAG (days after germination), root systems were measured using MRI. Plants were measured weekly for plant height and tiller number.

### MRI Measurements

**Figure 1:**
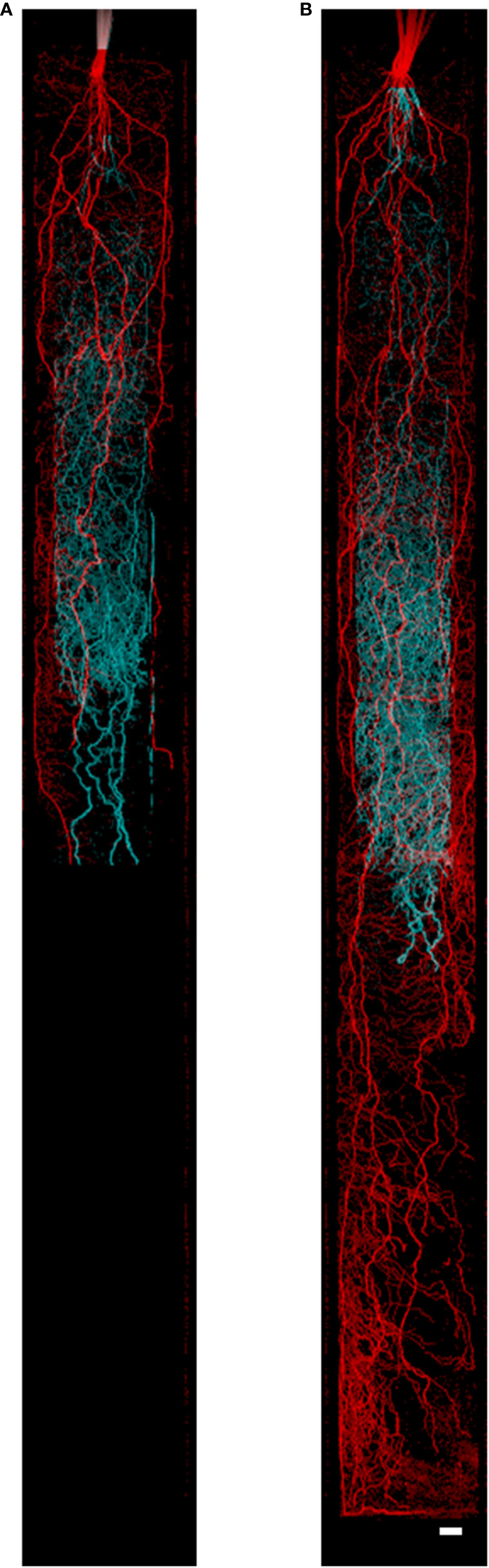
Nodal and seminal root systems as acquired by MRI at **(A)** 34 days after germination (DAG) and **(B)** 43 DAG. The seminal root system is represented in cyan growing in the inner cylinder, and the nodal root system is represented in red growing in the space between the inner and outer cylinder. Image **(A)** consists of five slice blocks of 96 slices (resolution 0.5 × 0.5 × 1 mm^3^), whereas **(B)** consists of nine slice blocks. Each slice block required 20 min of measurement time. To separate the seminal and nodal root systems, masks were constructed with the same size as the physical compartments. Plants were grown in 80 cm mesocosm. Scale bar = 1.5 cm.

MRI measurements were performed on a 4.7 T magnet (Magnex, Oxford, UK) with a Varian console (van Dusschoten et al., 2016). Root system architecture was measured according to protocols described in (van Dusschoten et al., 2016). In brief, the magnet has a vertical bore (310 mm diameter) and magnetic field gradient coils (205 mm diameter) that created gradients of up to 300 mT m^−1^ mm which allowed plants to be measured in an upright position. For the split root system experiment, once the rooting depth exceeded 50 cm, resolution was reduced from 0.5 × 0.5 × 1 mm^3^ to 0.75 × 0.75 × 1.5 mm^3^ due to measurement time constraints. In one instance, we measured a complete root system at full resolution as a reference (**Figure 1**). Using the MRI enabled the quantification of the accumulation of root length over time in the inner and outer compartments.

For split root system experiments, a light installation was built on top of the magnet using 20 W LED’s (warm white (2700K), Bridgelux BXRA-30E2000-B, USA). Mesocosms were placed on the top of the magnet before measurements, so it was important to keep environmental conditions constant to ensure no spurious environmental effects and root water uptake. The light intensity for the plants was maintained at 400 µE m^−2^s^−1^, and photoperiod was kept consistent with climate chamber experiments (14 h daylight).

For both experiments, at 19, 29, 34, and 43 DAG mesocosms were measured for root and soil water content. Initial soil water concentration at the beginning of each MRI measurement was kept between 0.093 and 0.113 µl mm^−3^ (*i.e.* below 12% soil water moisture). Twenty-four hours later, mesocosms were measured again for soil water content. Differences in soil water content between the first and second measurement points were calculated as soil water depletion. To measure soil water content, we used a three-dimensional spin echo sequence with a 0.8 ms echo time, in contrast to the 9 ms we use for root imaging. Soil water content was quantified with reference to a calibration curve (**Supplemental Figure 2**) that relates MRI signal intensity to gravimetrically determined soil water content. Soil water depletion can be quantified based on differences in soil water content over a 24 h period and can be determined locally with a resolution of 3 × 3 × 3 mm^3^. When we assume zero growth or anatomical changes in this 24 h period, the water in the roots that is included in the three-dimensional measurements is of no relevance. Twelve minutes is required to measure soil water content in a 6 cm image block (cylinder 6 cm × 8 cm). Due to time constraints, we measured soil water content in the split root system in six image blocks evenly distributed along the whole tube. Plants were watered approximately 12 h before measurements to 12% soil water content to allow the water to redistribute evenly throughout the mesocosm. To calculate root water uptake, the average hourly soil water depletion (average over a 24 h period) was divided by the total length of the root system, as measured by the MRI. The soil water redistribution over the separated soil compartments nets out to zero (integral over z = 0), and therefore root water uptake of seminal and nodal root systems can be measured. The nodal root system refers to the nodal axial roots and all attached laterals. The seminal root system refers to the seminal axial roots and all attached laterals.

### Plant Harvests

Upon the completion of each experiment, the plants were destructively harvested in order to evaluate detailed shoot characteristics (shoot dry weight and leaf area) and root architectural and anatomical phenotypes (number of xylem vessels, root cross-sectional area, root length, root cortical senescence). For all experiments, the entire shoot was excised and leaf area was measured (LI-3100 leaf area meter, Licor, Nebraska, USA).

#### Cylindrical Split-Root System Experiments

The outer compartment tube was removed from the setup. The roots growing into the inner compartment were excised at the base of the seed. The soil from the roots growing in the outer compartment was gently shaken off, and the roots were gently washed with low pressure water. The entire, in-tact nodal root system was preserved in 70% ethanol for root length analysis. Roots from the inner compartment were washed with low pressure water, and the entire root system was stored in 70% ethanol for root length analysis (**Supplemental Figure 1**). Root length was measured by scanning and analyzing preserved root segments using WinRHIZO Pro (Régent 389 Instruments, Québec City, Québec, Canada). Seminal and nodal root segments, 2 cm in length, were collected (seminal roots: 2, 6, 12, 18, 24, 30, and 36 cm from the root apex and nodal roots: 2, 3, 8, 13, and 18 cm from the root apex) from two roots per plant and were preserved in 70% ethanol for anatomical analysis.

#### Relating MRI Signal Intensity to Root Age Experiments

The soil and roots were removed from the mesocosm. The soil was gently shaken off the root system, and the roots were washed with low pressure water. The entire root system was stored in 70% ethanol for anatomical analysis. Anatomical analysis was performed on three seminal roots per plant. Seminal root segments, 2 cm in length, were collected at 2, 6, 12, 18, 24, 30, and 36 cm from the root apex.

### Image Data Analysis

Root length and root diameters were extracted from the MRI images using the NMRooting software described in van Dusschoten et al. (2016). In this software the root diameter is estimated by integrating the MRI signal intensity around a root segment. Pilot experiments demonstrated that changes in root anatomy such as aerenchyma formation or cortical senescence can reduce MRI root signal intensity, causing an apparent reduction of the root diameter. Therefore, in the current study, we refer to root diameter, which in reality is the effective root diameter as calculated by NMRooting based on signal intensity.

In the signal intensity experiment, the development of seminal root segments was monitored over 23 days. For the first measurement time point (DAG 12), we delineated all seminal roots for each plant from the root apex to the seed. In these segments the root diameters are calculated on each position, presented relative to the distance to the root apex, and averaged over all seminal root segments. The analyzed seminal root segment lengths ranged from 4 to 18 cm. Using the segments determined in the data from DAG 12, this analysis was then repeated for the following measurements to observe the diameter changes during root aging. Younger roots are visible in the MRI images and in later growth stages may be rendered ‘disappeared’ (*i.e.* were no longer visible) through a loss in root signal intensity. Disappeared roots are the ‘known’ disappeared roots, or roots that were once visible in the MRI. Changes in signal intensity over time in different genotypes were compared.

For the split root system, the MRI data from the inner and outer compartments were separated manually using digital, positional masks that followed the shape of the inner cylinder. Since the wall between the compartments was 4 mm wide, this allowed for inconsistencies of tube placement without affecting the separation of the compartments for further analysis. Calculations of root length were performed on the separated nodal and seminal root systems, the inner compartment containing the seminal root system, the outer compartment the nodal root system. Soil water content and its depletion over a 24 h period were calculated based on a reference to a calibration curve for each compartment. Water uptake per root length was compared between the seminal and nodal root systems; the ratio of seminal and nodal root lengths and root water uptake was analyzed over time. For all experiments, significance was determined at p < 0.05 and was determined through Tukey HSD tests. Two genotypes were used in the experiment that contrasts in root anatomical traits. No differences in root water uptake in seminal or nodal roots were observed between genotypes, so they were grouped for subsequent analysis.

Average root age in the seminal and nodal root systems was calculated as:

(1) $$\langle RA(t_{n})\rangle =\frac{\sum_{n=1}^{n=4}NRL(t_{n})\cdot (t_{n-1}+(t_{n}-t_{n-1})/2)}{TRL(t_{n})}$$

Where RA = Root age, NRL = New Root Length, TRL = Total Root Length, and t_n_ = 19,29,34,44 Days after Germination.

### Anatomical Characterization

Root segments preserved in ethanol were stained with acridine orange in order to visualize root anatomy. Acridine orange stains cell walls and viable cell nuclei. Acridine orange staining was performed according to the protocol of Henry and Deacon (1981); however, the staining time was extended to 30 min. Stained root segments were embedded in a gelatin capsule with Tissue-Tek CRYO-OCT compound (Fischer Scientific, Massachusetts, USA) and frozen at −20°C for 15 min. A Kryostat 2800 Frigocut –E (Reichert-Jung, Leica Instruments GmbH, Nussloch, Germany) was used to cut transverse sections 60 µm thick. Root cross sections were imaged on a compound microscope (Zeiss Axioplan 2, mounted with an AxioCam ICc 5, Filter 09: Blue 450-490 nm Carl Zeiss Jena GmbH, Jena, Germany; 20× magnification). Cross-sectional images were phenotyped for living root cortical area and area of the stele using ImageJ (Rasband, 2015). Root surface area was estimated as the root length multiplied by the average diameter of a seminal root or nodal root (**Table 1**). For data analysis, nodal and seminal roots from all genotypes were grouped (n = 4 for all experiments).

**Table 1:** Anatomical parameters of seminal and nodal roots at 35 DAG.

	Distance Behind the Apex (cm)	Stele Diameter (um)	Number of metaxylem vessels per root	Cross-section Diameter (um)	% Cortex Senesced	Diameter of metaxylem vessels (µm)	Axial Hydraulic Conductivity (L_x_ * 10^11^ [m^3^ s^−1^ mPA^−1^)]
**Nodal**	3	211	1.0	384	6.3	30	1.986
	8	204	1.6	358	12.0	30	5.233
	13	199	3.4	269	28.3	26	15.734
	18	215	3.1	254	32.1	26	12.640
**Seminal**	6	169	1.1	267	13.0	29	2.018
	12	160	1.0	241	22.1	29	1.816
	18	181	1.0	187	36.2	29	2.172
	24	171	1.1	161	43.2	31	2.471
	30	187	1.0	133	48.2	29	1.923
	36	176	1.1	155	46.7	30	2.419

Numbers presented are an average of four roots. Percent cortex senesced was calculated based on the difference in cross-sectional areas (total cross-sectional area minus stele area) at 3 cm from the root apex and at the sample position. Axial hydraulic conductivity was estimated using Hagen–Poiseuille’s law.

## Results

**Figure 2:**
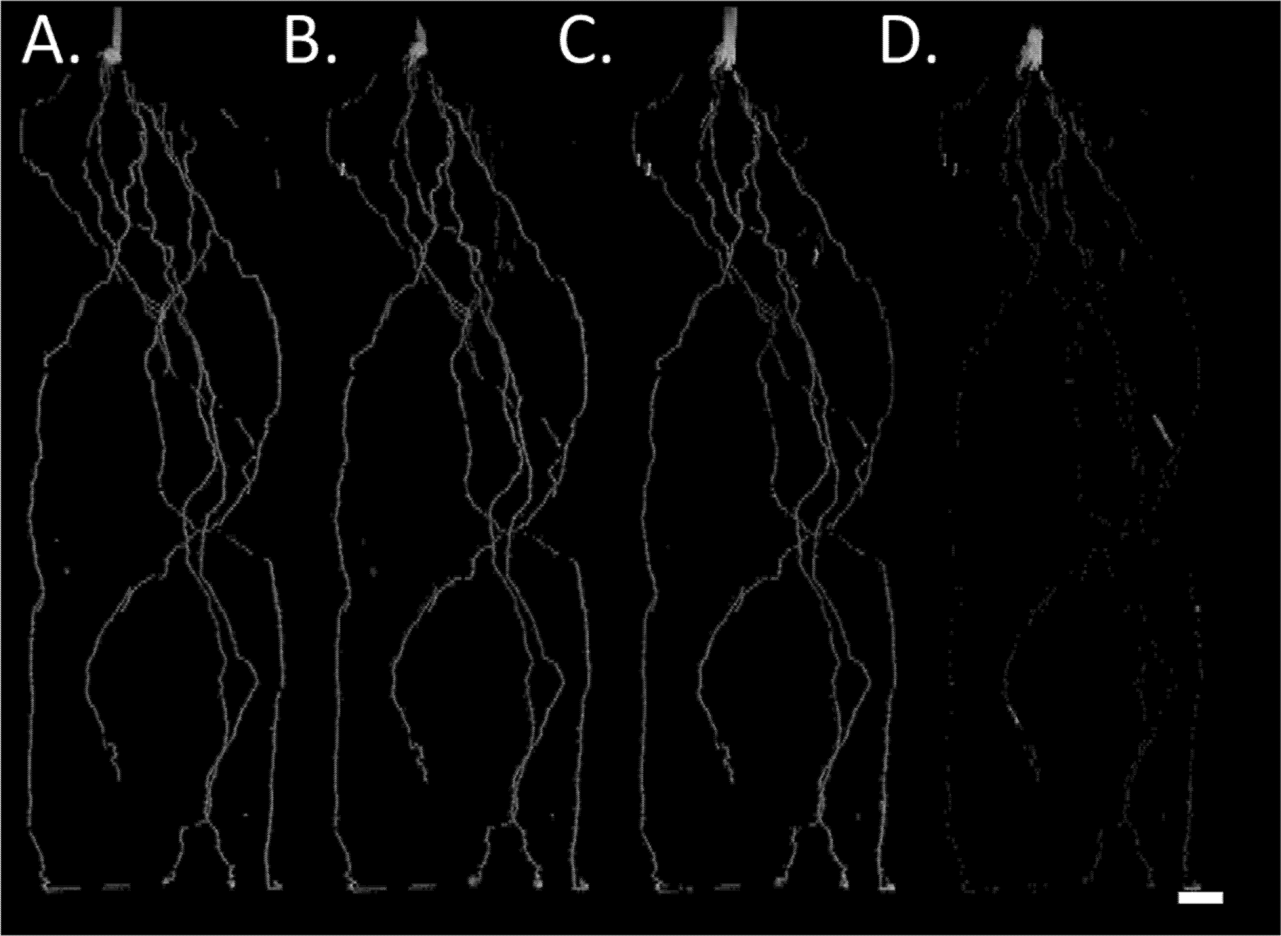
MRI signal intensity development of barley seminal root over time. **(A)** Complete root system at 12 DAG, **(B–D)** root system 19, 27, and 35 DAG; only roots already present at 12 DAG are shown. Plants were grown in a cylindrical mesocosm (30 cm × 8 cm). Reductions in root signal intensity occur in older root segments but not in for example, the stem which functions as max intensity reference. For image D the shoot was removed prior to the MRI experiment. Resolution 0.5 × 0.5 × 1.0 mm^3^, Scale bar = 2 cm.

In order to account for the varying root MRI signal throughout plant growth, we characterized and quantified root signal intensity changes to enable the accurate estimation of root length. Results demonstrated that aging of barley roots reduced the MRI root signal intensity in seminal roots (**Figure 2**) and in nodal roots (data not shown). In some cases, the loss in signal intensity in later growth stages was so severe that seminal roots could no longer be detected in MRI images (*e.g.* **Figure 2**, root diameter detection limit approximately 250 μm). Therefore, it is important to characterize and quantify root signal intensity changes for subsequent corrections in root length. In the first 5 cm behind the root apex (measured at every timepoint), the signal intensity of roots of both genotypes at 12, 19, and 27 days after germination (DAG) was not significantly different. At 35 DAG, the signal intensity in the first 5 cm behind the root apex was reduced by 59% compared to 12 DAG. At 5–20 cm from the root apex, the signal intensity of roots decreased by 12, 28, and 72% at 19, 27, and 35 DAG, respectively, compared to 12 DAG. At 20–35 cm from the root apex, the signal intensity of roots decreased by 13, 46, and 67% at 19, 27, and 35 DAG compared to 12 DAG. No significant differences in signal intensity were observed between genotypes at 12 and 35 DAG. In contrast, at 19 and 27 DAG, the signal intensity of Tkn24b was significantly reduced at 5–20 cm from the root apex compared to Arena. No significant differences were observed between genotypes at 19 and 27 DAG near the root apex (0–5 cm, 0% of original signal intensity lost) or in the proximal sections (20–35 cm, 13–46% of the original signal intensity lost). A strong negative correlation (R^2^ = 0.78, p < 0.05) was observed between distance from the root apex and root signal intensity at 12, 19, and 27 DAG. A weaker negative correlation (R^2^ = 0.34, p < 0.05) was observed between distance behind the root apex and root signal intensity at 30 DAG due to the loss of signal intensity along the entire root (**Figure 2**). This rate in signal loss is similar to the rate of root cortical senescence in seminal roots as determined by microscopy at 35 DAG, and the percentage of cortex senesced increased with distance behind the root apex (**Table 1**, **Supplemental Figure 3**).

MRI microscopy using a small, highly sensitive radiofrequency coil that was inserted into the soil facilitated the visualization of root cortical and stele tissues on small bundles of seminal roots (**Supplemental Figure 3**). These images indicate water loss in the cortex and epidermis with root age through a loss in MRI signal intensity. At 12 DAG seminal roots had a visible diameter between 600 and 700 µm. At 35 DAG, the cortex and epidermis were no longer visible in the MRI microscopy images; only stele tissue was visible and had a diameter between 150 and 250 µm (**Supplemental Figure 3**). Such fine roots are at the detection limit in the wider radiofrequency coil used in all other MRI experiments in this study. Root diameters and anatomy detected in the MRI at 35 DAG were confirmed with destructive harvests and root sectioning and staining methods (**Supplemental Figure 3**). Although various root aging processes may contribute to signal loss, we conclude that RCS is a major explanatory factor.

The loss of the root cortex and subsequent reductions in root diameter result in the reduction or complete loss of the root MRI signal as a signal threshold is used to discriminate root tissue from noise and small signal clusters stemming from soil water. Root diameters below approximately 250 µm are not detectable by the MRI, and therefore the majority of seminal root length at 35 DAG was not visible in the MRI image (**Figure 2**, **Table 1**). It is important to understand and quantify changes in root MRI signal intensity in order to interpret root length data evaluated by the MRI. Roots that were once visible in the MRI at younger growth stages may have disappeared in the image through a loss in root signal intensity. We refer to disappeared roots as roots that were once visible in the MRI. These insights regarding the loss of root signal intensity with root age were then used to better interpret root and water dynamics in subsequent experiments.

We developed a concentric split root system that physically and hydraulically separates the nodal and seminal root system in order to study these root systems independently. At 29 DAG, MRI-root-signal-intensity-changes began observing regions in seminal and nodal roots of Tkn24b and Arena genotypes that had disappeared from the image (**Supplemental Table 1**). Disappeared root length comprised 44% of the total root length (average of 15.6 m) in seminal roots compared to 49% of the total root length in nodal roots (average of 40.7 m) at 43 DAG. In both nodal and seminal roots the greatest increases in the fraction of disappeared root length were observed between 34 and 43 DAG (**Supplemental Table 1**).

**Figure 3:**
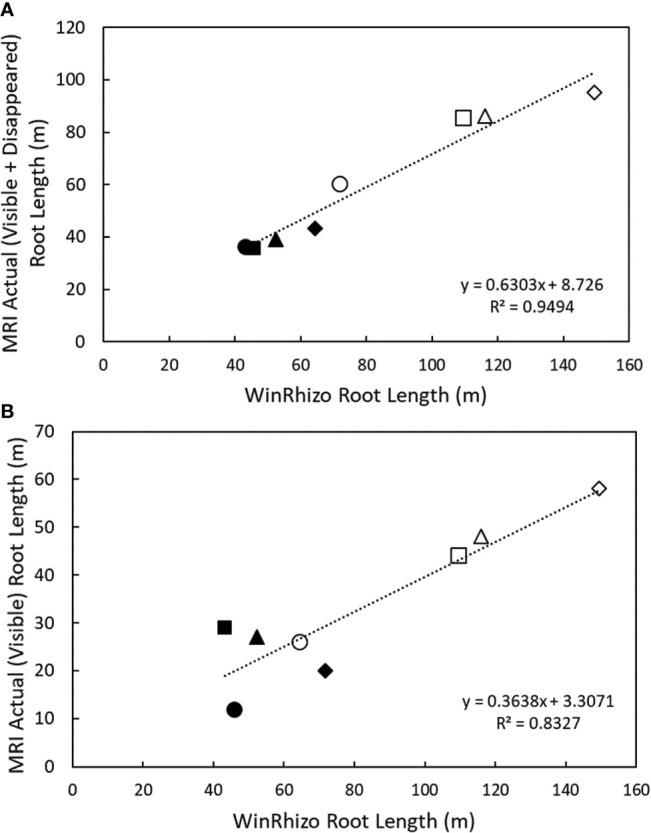
Correlation between nodal and seminal root lengths detected in the MRI and destructive harvest root length detected by WinRhizo at 38 DAG. **(A)** Correlation between cumulative root length (visible + disappeared) and **(B)** correlation between cumulative root length (visible) and root length measured in WinRhizo. Closed circles denote seminal roots, and open circles denote nodal roots. Plants were grown in a split root system (80 cm × 8 cm mesocosm) with physically separated nodal seminal root systems. Points (n = 4 for each root class, two replications using two genotypes) represent individual data points for each genotype, replication, and root class. Each symbol represents a different pot (*i.e.* matching symbols correspond to roots from the same pot).

At harvest, root length was measured destructively by WinRHIZO. WinRHIZO root length correlated more strongly with root length detected in the MRI after signal loss correction (visible + disappeared root length) (R^2^ = 0.95, y = 0.6303x + 8.726, p < 0.05, n = 8) compared to only visible root length in the MRI (R^2^ = 0.83, y = 0.3638x + 3.3071, p < 0.05). Total root length detected in the MRI was 75% of the root length measured by WinRHIZO (**Figure 3**, **Supplemental Table 1**), similar to van Dusschoten et al. (2016). All subsequent data analysis was performed on the total (visible + disappeared) MRI root length as it is a more accurate representation of root length measured by destructive harvests.

Using the concentric split root system, root length and water uptake over a 24 h period, could be quantified spatially and temporally and independently in seminal and nodal root systems up to 43 DAG. Using this same split root system configuration, we could detect up to 95 m of total root length of the nodal root system and up to 43 m of root length in the seminal root system for a single plant at 43 DAG. At 19 DAG, the nodal root system had started developing and was between 0.4 and 3.7 m in length. Between 19 and 34 DAG, the nodal root system accumulated length and root length density at a much faster rate compared to the seminal root system (**Supplemental Table 1**).

In addition, the seminal and nodal root systems explored different depth domains in the mesocosm. After 34 DAG, the nodal root system explored deeper soil domains compared to the seminal root system. After 34 DAG, the seminal and nodal root systems grew 56 cm day^−1^ and 17 cm day^−1^, respectively in total root length. However, between these time points, the nodal root system extended to the bottom of the 80 cm mesocosm and increased soil exploration by 40 cm in depth, while the seminal root system only extended to approx. 57 cm in depth which equated to an increase in only 11 cm in mesocosm depth (**Figure 1**, **Supplemental Table 1**).

Since redistributive soil water flows (as computed by the Richards equation for unsaturated water flow) can obscure local root water uptake, we integrated the soil water depletion rate over the whole mesocosm (**Supplemental Figure 3A**, the soil water depletion rate in zones absent of roots is comparable to that of zones with root occupancy and is caused by water transported upwards along the root system). No significant differences were observed in total water uptake per mesocosm for nodal or seminal roots except that total water uptake at 29 DAG in seminal roots was significantly less than other time points (**Supplemental Figure 4**). Therefore, we observed no large genotypic or environmental effects on total root water uptake.

**Figure 4:**
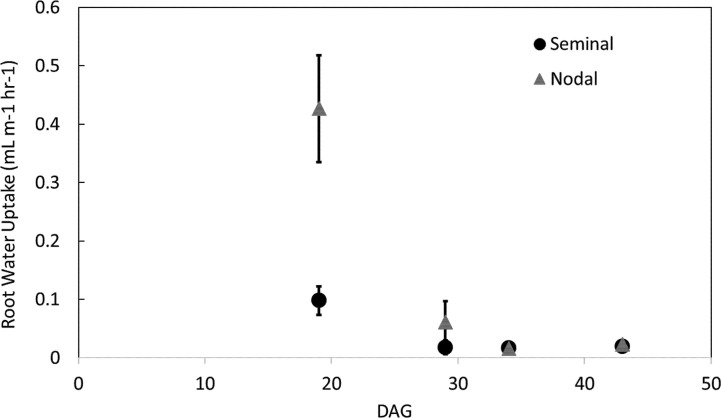
Water uptake per root length in seminal and nodal roots over time as determined by the MRI. Each data point represents the total water uptake per root class in one mesocosm at a specific time point. Plants were grown in a split root system in a mesocosm size of 80 cm × 8 cm. Points (n = 4 for each root class) represent the average of two genotypes in two replications. Genotypes were averaged as no difference in root water uptake was observed between genotypes.

Nodal root systems, however, had significantly greater water uptake per root length compared to seminal roots at younger plant growth stages (*i.e.* 19 and 29 DAG). At 19 and 29 DAG, nodal root systems had 79 and 59% greater water uptake per root length, respectively compared to seminal root systems. At 34 and 43 DAG water uptake per root length between nodal and seminal root systems was not significantly different (**Figure 4**). The same trends were observed in water uptake per root surface area. At 19 DAG, water uptake per surface area was 68% greater in nodal roots, and from 29 DAG onwards no significant differences were observed in water uptake per root surface area between seminal and nodal root systems (**Supplemental Figure 5**). Nodal root systems had greater variation in water uptake per root length compared to seminal root systems. In nodal root systems the greatest variability in water uptake per root length was observed at 19 DAG, and water uptake per root length varied eightfold among genotypes and replications. In seminal roots, the greatest variability in water uptake per root length was observed at 29 DAG, and water uptake per root length varied sixfold among genotypes and replications. Generally, variability in water uptake per root length decreased with plant age in both seminal and nodal roots (**Figure 4**).

Besides having greater water uptake per unit root length, nodal roots also had greater diameter, more xylem vessels, larger xylem vessels and consequently much greater computed axial conductivity. However, no large differences in variability of anatomical traits among seminal or nodal roots were observed (**Table 1**).

Throughout the duration of the experiment, the average seminal root system age increased from 9 to 12 days and for the nodal root system increased from approximately 3 to 10 days (Eq. [1]). Throughout the duration of the experiment, the ratio of root age between seminal and nodal root systems declined from 3 to 1.2. As the average age of the root system trends to the same age for the seminal and nodal root systems, so does water uptake per root length.

**Figure 5:**
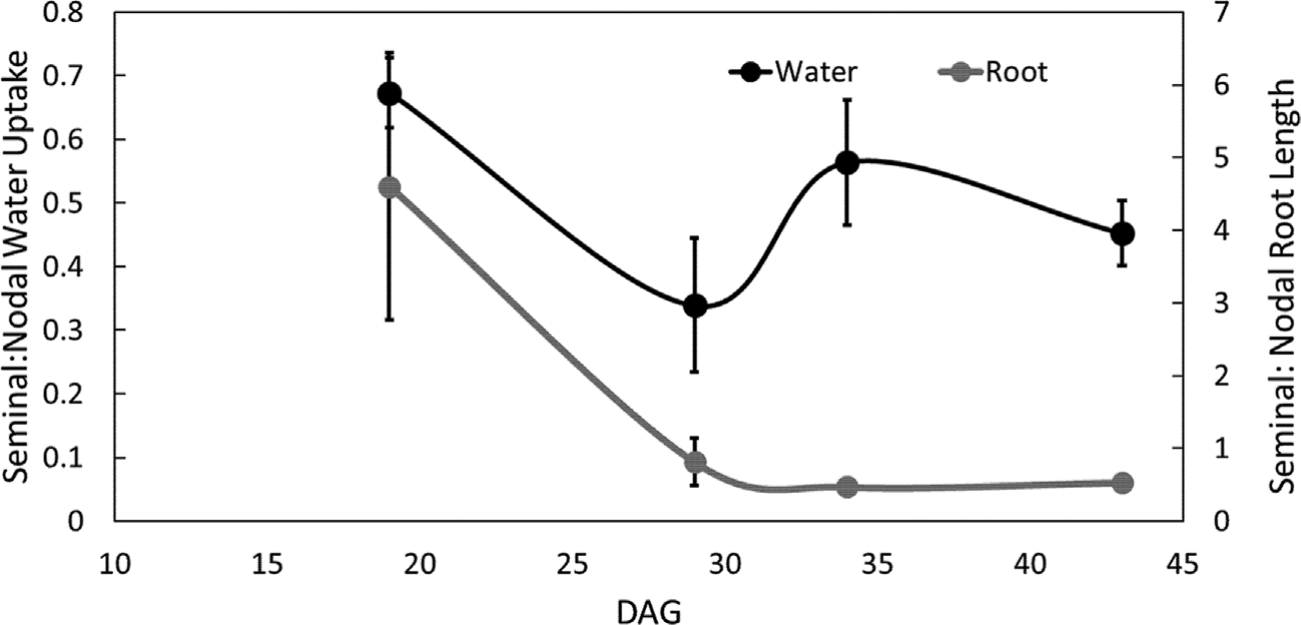
Ratio of seminal and nodal root length and water uptake over time. Each point is the ratio of root length or ratio of water uptake between seminal and nodal roots. Four individuals were grown in split root systems (mesocosm 80 cm × 8 cm) separating the nodal and seminal roots. Root length and water uptake were measured in the MRI up to 43 days after germination (DAG). Error bars represent the standard error. Points represent averages over all four individuals.

The ratio of seminal to nodal root length consistently decreased over time as the nodal root system of the main shoot and its tillers developed (**Figure 5**, **Supplemental Tables 1** and **2**). The ratio of water uptake between seminal and nodal root systems decreased by 54% between 19 and 29 DAG. At 29 DAG, the nodal root system was substantially larger in length and had a smaller fraction of disappeared roots compared to the seminal root system (**Supplemental Table 1**). Between 29 and 43 DAG, the ratio of seminal to nodal water uptake was not significantly different even though nodal root systems continued to accumulate substantially more root length compared to seminal roots (**Figure 5**, **Supplemental Table 1**).

## Discussion

Our results demonstrate that the seminal and nodal root systems do not develop in the same manner in terms of accumulation of root length, root length accumulation at depth, root length density, and water uptake (**Supplemental Table 1**, **Figure 5**). In young plants, water uptake per root length was greater in nodal root systems compared to seminal root systems. As plants matured, nodal root systems continued to take up more water than the seminal root system due to greater root length, but not greater water uptake per root length (**Figures 4** and **5**). At 43 DAG, the nodal root system accumulated more length and explored deeper soil domains compared to the seminal roots (**Figure 1**). Roots that explore deeper soil domains can improve plant fitness in conditions of drought or low nitrogen availability as mobile soil resources (*e.g.* water and nitrogen) are often located in deeper soil domains throughout the growth season due to leaching and evaporation from the topsoil (Lynch, 2013; Lynch, 2019).

In our study, in 19 DAG barley root systems grown in soil, the seminal root system made up the majority of the total root length and root surface area; however, the nodal root system contributed the majority of total water uptake. In a different study on barley, plants (14–17 d old) grown in solution culture, the seminal root system contributed 92% of the water uptake and comprised ~98% of the total root surface area compared to the nodal root system (Knipfer and Fricke, 2011). Knipfer and Fricke (2011) attribute the smaller contribution of the nodal root system to water uptake due to a smaller root surface area, a reduced number of developed axial roots, and smaller axial hydraulic conductivity per root surface area of nodal roots compared to seminal roots. Here, we also observe a smaller proportion of water uptake per root length and root surface area in nodal roots compared to seminal roots in early growth stages (*i.e.* 19 DAG).

In younger plants (*i.e.* 19 and 29 DAG), nodal root systems had greater water uptake per root length compared to seminal roots. This may be because the nodal root system, including their lateral roots, generally has larger diameters and therefore in this study, a 40% greater surface area per root length. Recent studies did not detect a significant difference between the radial hydraulic conductivity of barley nodal and seminal roots per unit surface area (Knipfer and Fricke, 2011; Schneider et al., 2017b). In addition to differences in root diameter, nodal and seminal roots have many other anatomical differences. Nodal roots of barley generally have increased metaxylem vessel size (Knipfer and Fricke, 2011; Tai et al., 2016) and number (this study) (Tai et al., 2016) and more cortical cell files compared to seminal roots (Knipfer and Fricke, 2011). In the current study, most regions of nodal roots had an increased estimated axial hydraulic conductivity compared to seminal roots due to a greater metaxylem vessel number and presumably have a greater radial hydraulic conductance due to a greater cross-section diameter (**Table 1**). In addition, nodal roots emerge at a later growth stage and are therefore younger than seminal roots, which emerge directly from the seed at germination. This may result in differences in the function and development of the endodermis as the endodermis in seminal roots is more developed (*i.e.* impregnated with more suberin and lignin in cell walls) than the endodermis of younger nodal roots and may be a more effective barrier for water and solutes (Robards et al., 1973; Enstone et al., 2003). However, water uptake in the soil depends on a variety of factors including changes in the apoplastic and cell-to-cell pathways, reduced root–soil contact, and increased endodermal suberization after the development of root cortical senescence (Schneider et al., 2017b; Schneider and Lynch, 2018) which are influenced by the growth environment and root class.

In young plants, nodal roots had significantly greater water uptake per root length compared to seminal roots. However, beginning at 24 DAG, there is no difference in water uptake per root length between nodal or seminal root systems (**Figure 4**). This difference in water uptake between root classes in younger plants (*i.e.* 19 and 29 DAG) could be due to differences in root age. The seminal root system emerges approximately two weeks before the nodal root system, and therefore water uptake may be significantly reduced due to aging processes, including the development of root cortical senescence. Throughout the duration of the experiment, the ratio of root age between seminal and nodal root systems declined from 3 to 1.2 (data not shown). As the average age of the root system trends to the same age for the seminal and nodal root systems, so does water uptake per root length. We speculate that root aging processes, including the development of root cortical senescence, play a significant role in root water uptake.

In the current study, the length of the nodal root system was much greater than that of the seminal root system (**Supplemental Table 1**). At 43 DAG, nodal root length was between 41 and 57% greater than seminal root length. Seminal and nodal roots had similar lateral branching densities and lengths (data not shown). The greater surface area of nodal roots per unit length increased metaxylem vessel size, and younger root tissue (in earlier growth stages) compared to seminal roots could contribute to greater root water uptake in nodal roots compared to seminal roots.

Root cortical senescence involves programmed cell death of cortical tissues. Previous studies have demonstrated root cortical senescence to be a common phenomenon in barley grown in soil and solution culture (Schneider et al., 2017b; Schneider et al., 2017a; Schneider et al., 2018). Previous studies using traditional sectioning and microscopy yielded similar results to ours and demonstrated that in seminal root segments at 30 DAG, the majority of the cortex and epidermis had senesced, and only the stele was visible, with a diameter between 140 and 225 µm (Schneider et al., 2017b). On average, the stele is 180 µm in diameter in seminal roots and 210 µm in diameter in nodal roots grown in soil, which is below the detection limit of the normal root imaging MRI (this study; van Dusschoten et al., 2016; Schneider et al., 2017b). As root cortical senescence develops, and the root cortex is lost, root diameter (or effective root diameter as measured by the MRI) may significantly decrease, causing a strong reduction in the MRI signal and in many cases causes the signal to become less than our noise threshold. We conclude that root cortical senescence is likely to cause MRI signal loss, and that the pattern of observed signal loss is similar to the pattern of root cortical senescence formation in barley.

Root anatomical changes and root age may not only influence the MRI signal intensity, but also influence root water uptake. To our knowledge, we are the first to present the importance of MRI signal intensity changes in the context of plant roots which are important considerations for future studies. In the current study, we demonstrated that the loss in MRI root signal intensity is important to consider when studying root dynamics and enabled us to better use root length data as acquired by MRI. Root length evaluated through WinRHIZO correlated more strongly with total (visible + disappeared) root length compared to only visible root length (**Figure 3**). Previous studies demonstrated that the development of root cortical senescence is accompanied by a severe reduction in root radial hydraulic conductivity (Schneider et al., 2017b). The most likely reason for these discrepancies in water uptake and the development root cortical senescence may be due to the role of lateral roots in water uptake. This study did not distinguish axial root length and lateral root length as the dense accumulation of root length and overlapping roots made it difficult to define appropriate segmentation criteria for the NMRooting software. The diameter of many lateral roots are below the detection limit of the MRI (here we estimate 25% of the root length is below the detection limit of the MRI) (**Figure 3**). In addition, lateral and axial root tissues cannot be distinguished by root diameter alone as the development of root cortical senescence significantly reduces apparent root diameter. However, previous studies have demonstrated that lateral roots comprise the majority of root length and are responsible for the majority of nutrient capture (Schneider et al., 2017a). It has been reported that lateral roots do not manifest root cortical senescence (Henry and Deacon, 1981; Schneider et al., 2017a) and therefore might not display reduced radial hydraulic conductivity over time. After the development of root cortical senescence in axial roots, we speculate that lateral roots perform the majority of root water uptake. Other previous studies have also demonstrated that lateral roots play a major role in root water uptake. Ahmed et al. (2016) demonstrated that in 16 day old maize plants water uptake by lateral roots was substantially greater than that of seminal roots. In the current study, it is not possible to evaluate the effects of root cortical senescence on root water uptake due to the confounding effects of lateral roots. So, even though the radial conductivity of seminal and nodal root systems is negatively affected by root cortical senescence (Schneider et al., 2017b), there appears to be no overall effect of root cortical senescence on total water uptake by the seminal or nodal root system as lateral roots presumably comprise the majority of the root length compared to the axial roots.

MRI signal intensity decreased with root age (**Figure 2**), and the loss in root signal intensity occurred in predictable patterns spatially and temporally on a root. The MRI signal of the root is related to the number of protons present in the root, the mobility of protons, and air–water interfaces inside the root. The senescence of root cortical tissue and consequent loss of water and protons in the root cortical tissue may cause the reduction or disappearance of the MRI root signal intensity. However, air pockets within the roots cause differences in the magnetic susceptibility and thereby cause local non-linear magnetic field gradients. These indirectly reduce the protons’ MRI signal near the air pockets and so enhance the signal loss (an air pocket directly reduces the signal). As cells in the roots are typically large compared to the diffusional pathway within 10 ms (less than 10 μm), changes of the cell sizes would have minimal effects on the MRI/NMR signal unless these are drastic. Cell leakages would have an undetermined effect on the MRI signal intensity. The development of root cortical aerenchyma would also result in a loss of cortical tissue and decrease root signal intensity; however, aerenchyma did not develop in plants studied here. In the current study, signal intensity changes resulted in the disappearance of up to 63 m in root length in the MRI image, which we partially attribute to the development of root cortical senescence and which has implications for analysis and interpretation of results.

Differences in water uptake between seminal and nodal root systems were observed and these differences were related to age (**Figure 2**, **Supplemental Figures 3** and **6**), and we assume age-related anatomical changes, including the development of root cortical senescence and increased metaxylem vessel number. Our split root system was successfully used to separate the seminal and nodal root systems resulting in the capability to evaluate these root classes and their water uptake independently and without interference of soil water flows. To our knowledge this is the first study that was able to spatially and temporally track the development of more than 135 m of root length, attribute them to root class and subsequent water uptake *in situ*. Characterization of root signal intensity in the MRI enabled us to correct for disappeared root length in the MRI image. This split root system method can also be used with maize, bean, other grasses, or any root system with spatially distinct root classes. The development of methods to understand the spatial and temporal dynamics of root system development and water uptake is important to understand plant function and adaptation and for the development of optimized irrigation systems. Factors that influence water uptake, how water uptake changes over plant growth, and the relative contribution of water uptake by individual root classes are important questions that can be addressed through the use of a split-root system in the MRI. Here we demonstrate that MRI can be used to quantify root and water dynamics of soil-grown barley. MRI technology provides unprecedented possibilities to study root water uptake *in situ* and three-dimensionally. Many factors contribute to root water uptake including root class and anatomy and understanding the spatial and temporal dynamics of root water update is important for the study of plant function and adaptation. Since nodal roots perform the majority of water uptake, nodal root phenes merit consideration as a selection target to improve water capture in barley and possibly other crops.

## Data Availability Statement

The datasets generated for this study are available on request to the corresponding author.

## Author Contributions

HS and DD designed and performed the experiments. JK, DP, JP, and DD developed the methodology, equipment, and data analysis. All authors contributed to the article and approved the submitted version.

## Funding

This project was institutionally funded by the Hemholtz Association. Plant material was provided by IPK Gatersleben. DP was financed by the German Plant Phenotyping Network (DPPN) funded by the German Federal Ministry of Education and Research (project identification number: 031A053).

## Conflict of Interest

The authors declare that the research was conducted in the absence of any commercial or financial relationships that could be construed as a potential conflict of interest.
