# Supplementary material for: Spatio-Temporal Variation in Water Uptake in Seminal and Nodal Root Systems of Barley Plants Grown in Soil
Source: Front Plant Sci. 2020 Aug 13;11:1247. doi: 10.3389/fpls.2020.01247 (PMC7438553; doi:10.3389/fpls.2020.01247)
Supplement: Supplementary file 1 [file DataSheet_1.pdf]

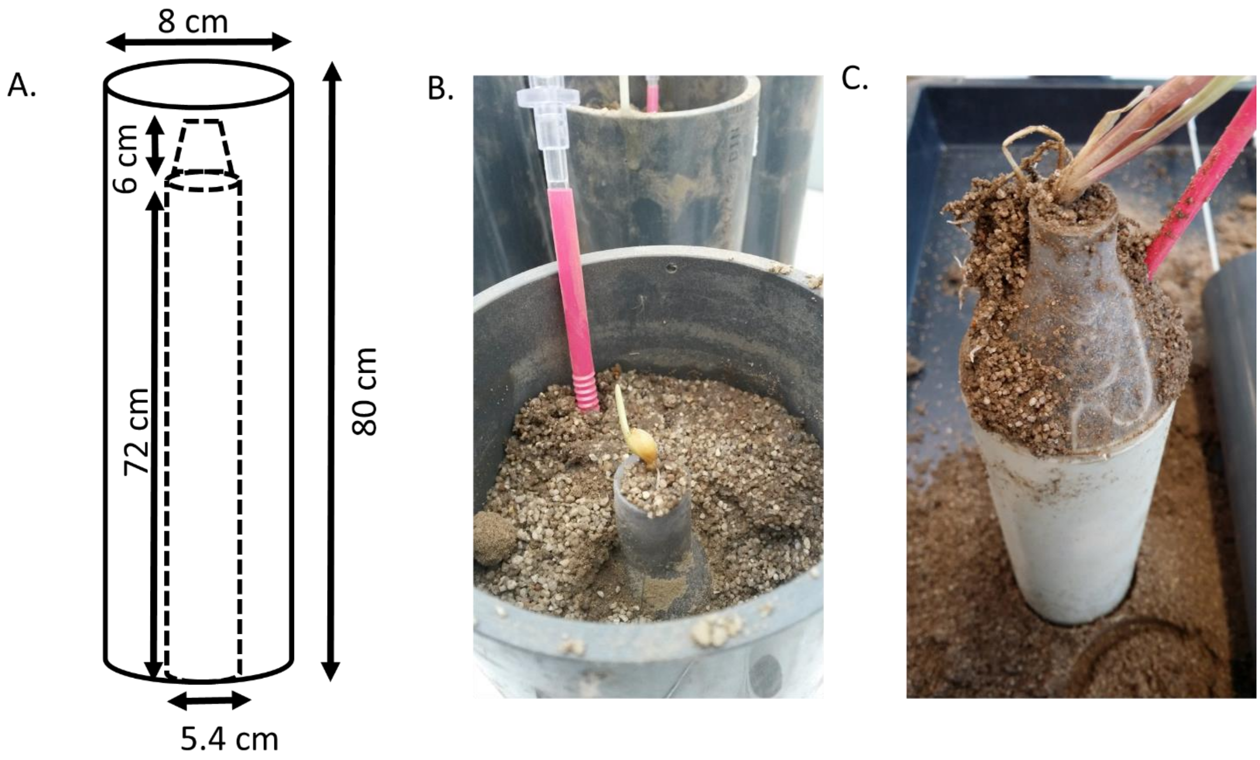

Supplemental Figure 1. Concentric split root system setup. A) schematic design of split root system. B) An image of the germinated seedling at transplanting. The seed was planted just above the seminal root compartment so that the seminal roots grew down into the inner compartment. The nodal roots emerged above the seed and grew in the outer compartment. C) An image of the split root system at harvest. The nodal roots are located in the outer compartment and the seminal roots are located in the inner compartment.

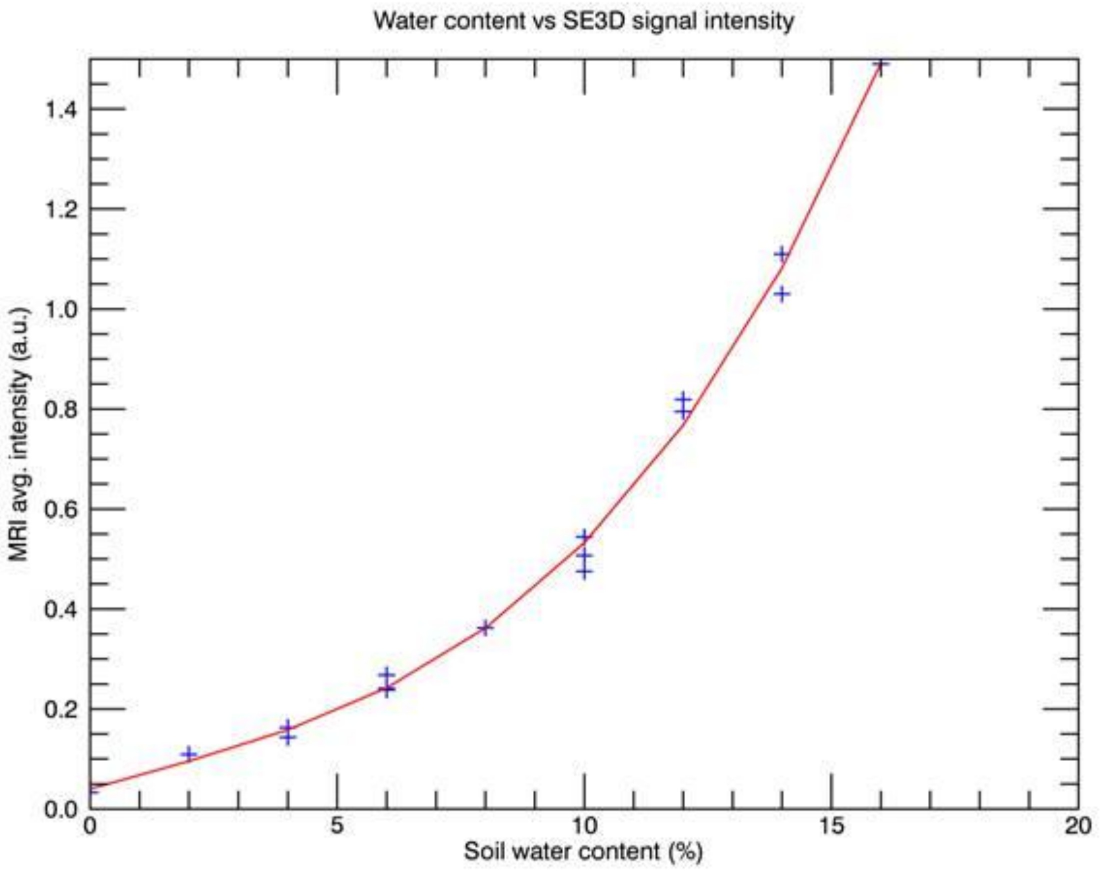

Supplemental Figure 2. MRI signal intensity vs gravimetric soil water content calibration curves. The water contents in the compartments ranged from 9-12%.

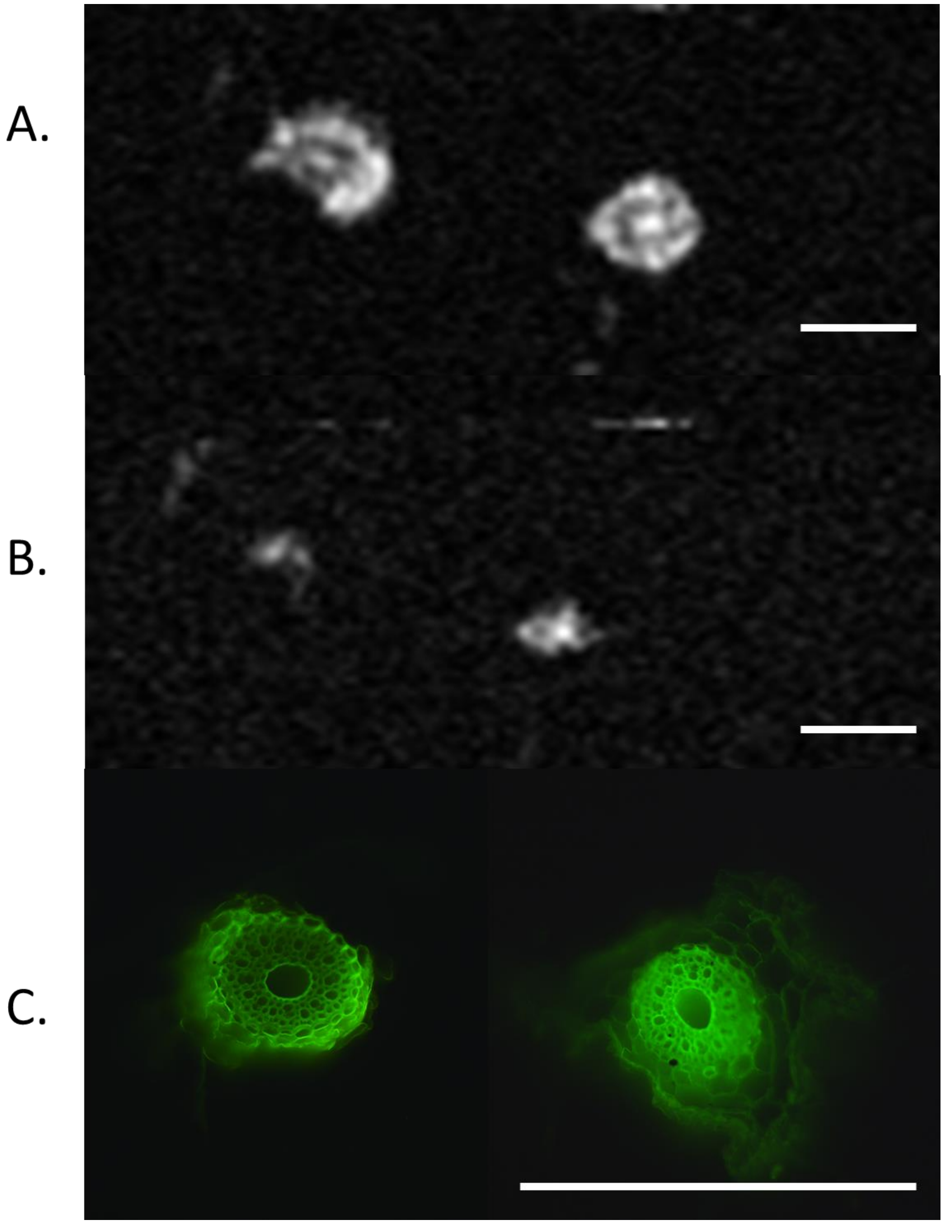

Supplemental Figure 3. MRI microscopy images of seminal roots as they change over time over time and light microscopy images of the same roots. A) Two seminal roots of Tkn24b imaged at 7 DAG B) Two seminal roots of Tkn24b imaged at 35 DAG (same root segments as A). C) Cryo-sectioning and staining of seminal root segments harvested at 35 DAG (same root segments as A and B). Resolution for A and B is 0.05 x 0.05 x 0.4 mm^3. 30 min MRI measurement time. Field of view 5 mm. Scale bars = 600 µm.

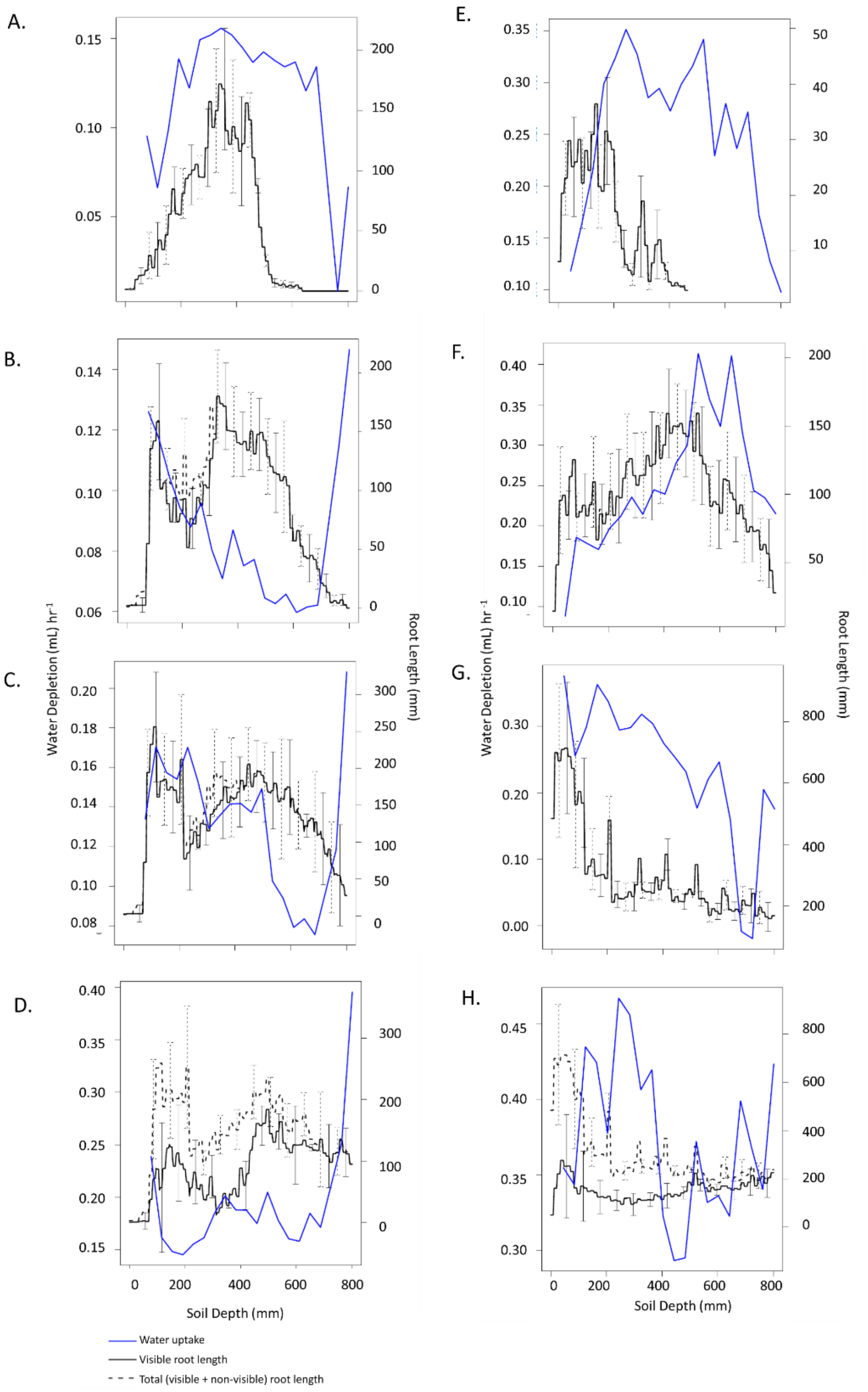

Supplemental Figure 4. Local water depletion and root length over a 24h period in seminal and nodal roots over time. Seminal roots at A) 19 DAG B) 29 DAG C) 34 DAG D) 43 DAG and nodal roots at E) 19 DAG F) 29 DAG G) 34 DAG H) 43 DAG. Error bars represent the standard error. Lines are the average of two replications of two genotypes. Plants were grown in a split root system (mesocosms 80 cm x 8 cm).

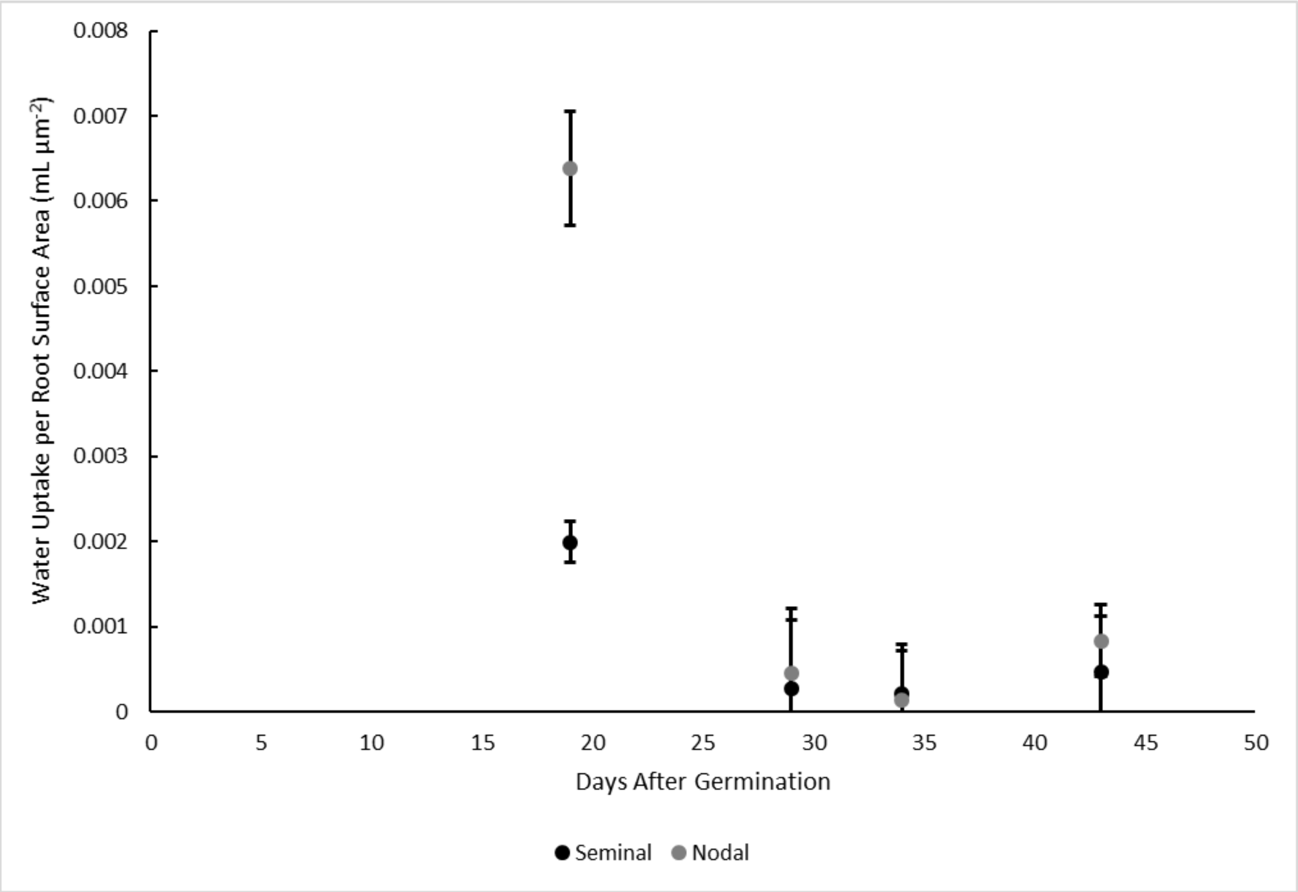

Supplemental Figure 5. Water uptake per root surface area in seminal and nodal roots over time as determined by the MRI. Each data point represents the total water uptake per root class in one mesocosm at a specific time point. Plants were grown in a split root system in a mesocosm size of 80 cm x 8 cm. Root surface area was estimated based on average root diameter for seminal and nodal roots. Points represent the average of two genotypes in two replications.

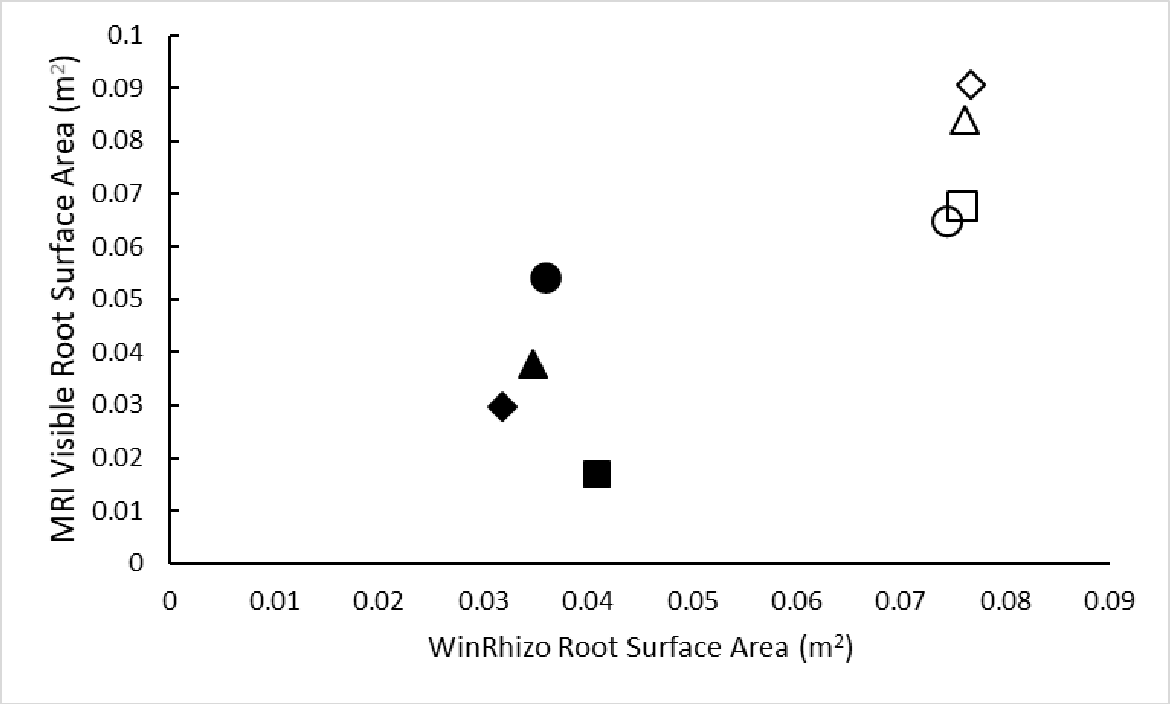

Supplemental Figure 6. Correlation between nodal and seminal root surface area detected in the MRI and destructive harvest root length detected by WinRHIZO at 38 DAG. Plants were grown in a split root system (80 cm x 8 cm mesocosm) with physically separated nodal and seminal root systems. Points (n=4 for each root class, 2 replications using 2 genotypes) represent individual data points for each genotype, replication, and root class. Each symbol represents a different pot (i.e. matching symbols correspond to roots from the same pot).
