# Supplementary material for: Spatio-Temporal Variation in Water Uptake in Seminal and Nodal Root Systems of Barley Plants Grown in Soil
Source: Front Plant Sci. 2020 Aug 13;11:1247. doi: 10.3389/fpls.2020.01247 (PMC7438553; doi:10.3389/fpls.2020.01247)
Supplement: Supplementary file 2 [file DataSheet_2.pdf]

Supplemental Table 1. Root and water uptake parameters detected through the MRI and destructive measurements. Each point is the average of two replications of two genotypes (n=4). Total root length (RL) is the sum of visible and disappeared root length in the MRI. WinRHIZO (WR) root length was measured at destructive harvests at the end of the experiment. Root and water dynamics were measured up to 43 days after germination (DAG).

Root Class	DAG	Total RL (m)	Disappeared RL (m)	Visible RL (m)	Root Length Density (cm cm^{-3})	Water Uptake (ml)	WR Root Length (m)
Nodal	19 DAG	2.25	0	2.235	0.08	23.305	-
	29 DAG	27.5	0.07	27.435	1.19	23.555	-
	35 DAG	80.1	0.05	80.015	3.70	23.84	-
	43 DAG	81.75	40.085	41.67	1.44	28.75	111.76
Seminal	19 DAG	7.15	0	7.155	0.36	17.58	-
	29 DAG	18.2	1.4	16.815	1.16	7.48	-
	35 DAG	33	1.72	31.26	2.81	12.505	-
	43 DAG	38.6	15.615	22.985	1.58	16.34	51.475

Supplemental Table 2. Plant growth parameters of the split root system experiment. Data was collected on two genotypes (arena and Tkn24b) up to 43 days after germination (DAG). Data shown are means (n=4).

DAG	Tiller #	Height (cm)	Leaf Area (cm2)	Shoot Dry Weight (g)
19	1	15.9	-	-
29	3	26.7	-	-
34	3.5	31.8	-	-
43	4.5	42.75	101.2	0.4
